# Oxidative damage to urinary proteins from the GRMD dog and mdx mouse as biomarkers of dystropathology in Duchenne muscular dystrophy

**DOI:** 10.1371/journal.pone.0240317

**Published:** 2020-10-08

**Authors:** Jessica R. Terrill, Basma A. Al-Mshhdani, Marisa N. Duong, Catherine D. Wingate, Zahra Abbas, Angelo P. Baustista, Amanda K. Bettis, Cynthia J. Balog-Alvarez, Joe N. Kornegay, Peter P. Nghiem, Miranda D. Grounds, Peter G. Arthur

**Affiliations:** 1 School of Molecular Sciences, The University of Western Australia, Perth, Australia; 2 Department of Veterinary Integrative Biosciences, College of Veterinary Medicine and Biomedical Sciences, Texas A&M University, College Station, Texas, United States of America; 3 School of Human Sciences, The University of Western Australia, Perth, Australia; Emory University, UNITED STATES

## Abstract

Duchenne muscular dystrophy (DMD) is a lethal, X-chromosome linked muscle-wasting disease affecting about 1 in 3500–6000 boys worldwide. Myofibre necrosis and subsequent loss of muscle mass are due to several molecular sequelae, such as inflammation and oxidative stress. We have recently shown increased neutrophils, highly reactive oxidant hypochlorous acid (HOCl) generation by myeloperoxidase (MPO), and associated oxidative stress in muscle from the GRMD dog and mdx mouse models for DMD. These findings have led us to hypothesise that generation of HOCl by myeloperoxidase released from neutrophils has a significant role in dystropathology. Since access to muscle from DMD patients is limited, the aim of this study was to develop methods to study this pathway in urine. Using immunoblotting to measure markers of protein oxidation, we show increased labelling of proteins with antibodies to dinitrophenylhydrazine (DNP, oxidative damage) and DiBrY (halogenation by reactive oxidants from myeloperoxidase) in GRMD and mdx urine. A strong positive correlation was observed between DiBrY labelling in dog urine and muscle. A strong positive correlation was also observed when comparing DNP and DiBrY labelling (in muscle and urine) to markers of dystropathology (plasma creatine kinase) and neutrophil presence (muscle MPO). Our results indicate the presence of neutrophil mediated oxidative stress in both models, and suggest that urine is a suitable bio-fluid for the measurement of such biomarkers. These methods could be employed in future studies into the role of neutrophil mediated oxidative stress in DMD and other inflammatory pathologies.

## Introduction

Duchenne muscular dystrophy (DMD) is a lethal, X-chromosome linked muscle disease affecting about 1 in 3500–6000 boys worldwide [1, 2]. DMD is characterised by severe and progressive muscle wasting caused by mutations in the *DMD* gene and loss of functional dystrophin protein. In skeletal muscle, a lack of dystrophin results in increased susceptibility of the sarcolemma to contraction induced damage and subsequent myofibre necrosis [3–5]. Repeated cycles of widespread myofibre necrosis and progressive failure of regeneration over time leads to replacement of myofibres by fatty and fibrous connective tissue, with loss of muscle mass and function, leading to premature death of DMD boys [1, 5, 6].

While the exact mechanisms for myonecrosis and loss of muscle function in DMD are not fully understood, perturbed intracellular calcium homeostasis, inflammation and oxidative stress are implicated [7–9]. Proposed sources of oxidants in dystrophic muscle include mitochondria, inflammatory cells, NAD(P)H oxidase, xanthine oxidase, and decoupling of nitric oxide synthase (NOS) (via dislocation or translocation of nNOS from the dystroglycan complex of the sarcolemma) [10]. Notably, neutrophils contain the heme protein myeloperoxidase (MPO), which can oxidise chloride, thiocyanate, iodide or bromide in the presence of hydrogen peroxide to form highly reactive oxidants, with hypochlorous acid (HOCl) as the predominant physiological oxidant [11, 12]. We have shown increased neutrophil content, HOCl generation and associated oxidative stress in muscle from the GRMD dog and mdx mouse models for DMD [13, 14]. The mdx mouse is the most commonly used animal model for DMD but has a mild pathology, possibly due to its very short growth phase and lifespan, as well as the small size of mdx mice [3, 15, 16]. By contrast, the GRMD dog model manifests a more severe dystropathology with a rapidly progressing and fatal disease similar to DMD boys [17].

Oxidation of amino acid side chains or cleavage of peptide bonds by HOCl yields carbonyl derivatives in many inflammatory disorders such as chronic lung disease, inflammatory bowel disease, rheumatoid arthritis and sepsis [18, 19]. Measurement of these derivatives with the protein carbonyl assay provides a biomarker of irreversible protein oxidative damage [20]. Increased carbonylation of muscle proteins has been demonstrated by our laboratory in mdx and GRMD muscle and by others in DMD patients and the sarcoglycanopathy and dysferlinopathy dystrophies [13, 21–24]. Taken together, these data suggest that oxidative damage to proteins has a role in dystropathology, leading us to theorise that HOCl generation by neutrophils is a major cause of protein oxidation in dystrophic muscle.

To further test the role of neutrophil mediated oxidative stress in dystropathology, and to better evaluate drug treatments that target this pathway, we have been investigating biomarkers for HOCl derived oxidants in muscle and biofluids from animal models of DMD. Urine is particularly appealing because it can be obtained non-invasively, making longitudinal analysis practical, for instance using metabolic cages. Additionally, urine proteins are relatively stable and are derived from a variety of sources, including glomerular filtration of blood plasma, cell sloughing, apoptosis, and secretion of exosomes by epithelial cells [25]. A recent proteomic analysis of urine from DMD patients showed a dramatic increase in titin fragments, providing a potential a non-invasive test for pre-screening of muscular dystrophies [26–28]. Likewise, the development of urinary biomarkers for oxidative stress could have a role in measuring disease progression in DMD patients.

Carbonylation of proteins in urine has been shown from humans after competing in super-marathons or undertaking over-training resistance protocols and for patients with chronic renal failure [29–31]. In keeping with these studies, we utilised an immunoblotting technique, whereby carbonyls can be detected using antibodies against dinitrophenylhydrazine (DNP), a reagent that specifically reacts with protein carbonyl groups [32]. Immunoblotting is sensitive, can be performed by most laboratories, and can be modified for high throughput, enzyme-linked immunosorbent assay (ELISA) to detect carbonyls on albumin treated with as little as 20 nmol of HOCl per milligram of protein [33]. Despite the formation of carbonyls in the presence of HOCl, and the high correlation of carbonyl formation and MPO content in a variety of conditions, increased protein carbonylation cannot be taken as specific evidence of HOCl or neutrophil involvement, as a variety of oxidative mechanisms lead to their formation [34]. This lack of specificity is compounded by the instability of HOCl instability, necessitating indirect assays. One technique which overcomes these limitations involves measuring chlorinated tyrosine using isotope dilution liquid chromatography with mass spectrometry [13]. However, this technique does not identify the proteins targeted by HOCl, which if identified would permit a better understanding of their potential to be useful biomarkers using immunoassay technologies.

In the present study we aimed to establish whether proteins were oxidised in urine from dystrophic animals. Protein carbonylation was measured with an antibody to DNP with reactive oxidants generated by myeloperoxidase measured with an antibody to dibromo-tyrosine (DiBrY). The DiBrY antibody is specific for protein tyrosine halogenation, and has been shown to detect chlorination by HOCl or hypobromous acid (HOBr) [35–38]. To establish whether similar proteins were oxidised across species, we measured oxidised proteins in urine, plasma and muscle from the mdx and GRMD models of DMD. Using correlation analysis in dogs, the relationship between protein oxidation in muscle and urine was measured, as was relationship between protein oxidation (in both muscle and urine) and plasma creatine kinase (CK, an established marker of dystropathology), and muscle MPO (to measure neutrophil presence).

## Materials and methods

### Animal procedures

Animal care was governed by Texas A&M animal use protocol 2018–0182 (Standard Operating Procedures-Canine X-Linked Muscular Dystrophy) and principles outlined in the National Research Council’s Guide for the Care and Use of Laboratory Animals. Six GRMD (heterozygous male-4, homozygous female-2; 8 months to 6 years of age) and 6 normal dogs (male-4, Female-2, 6 months to 2 years) were evaluated. Studies from this same cohort of dogs have previously been published [39]. Dogs were fed LabDiet Advanced Protocol High Density Canine dry or wet food (PMI Nutrition). Urine was collected by either cystocentesis or by free-catch. Blood was withdrawn from the jugular or cephalic vein and then centrifuged to allow plasma removal for subsequent freezing at -80°C until analysis. Vastus lateralis muscle biopsies were taken under aseptic surgical technique at Texas A&M, immediately snap frozen in liquid nitrogen for immunoblotting, stored at -80 C, and sent to Perth for analysis.

Mouse experiments were carried out on dystrophic mdx (C57Bl/10ScSn-*Dmd*/^mdx^) and non-dystrophic control C57 (C57Bl/10ScSn) mice (the parental strain for mdx). All animal experiments were conducted in strict accordance with the guidelines of the National Health and Medical Research Council Code of practice for the care and use of animals for scientific purposes (2004), and the Animal Welfare Act of Western Australia (2002), and were approved by the Animal Ethics committee at the University of Western Australia. All mice were obtained from the Animal Resource Centre, Murdoch, Western Australia. Mice were maintained at the University of Western Australia on a 12-h light/dark cycle, under standard conditions, with free access to food and drinking water. Equal numbers of male and female mice were used for analysis. Mice were placed in metabolic cages for 24 hours prior to sampling to collect urine. All mice were sacrificed at 6 weeks by cervical dislocation while under terminal anesthesia (2%v/v Attane isoflurane). Whole blood was collected via cardiac puncture followed by centrifugation, plasma removal, and freezing at -80°C until analysis. Quadriceps muscles were collected and immediately snap frozen in liquid nitrogen for immunoblotting.

### Protein extraction and immunoblotting

Before analysis was performed on all animal samples, the immunoreactivity of the antibodies to protein treated with HOCl, as well as two other common oxidants, hydrogen peroxide and hydroxyl radical, was tested. Normal dog plasma was incubated with and without 1 mM of each oxidant, and immunoblots performed, as described below. As shown in S1, signal for both the DNP and DiBrY in dog plasma was increased with HOCl exposure, with little to no additional labelling occurring after exposure to hydrogen peroxide and hydroxyl radicals.

Mouse urine was not processed before analysis. However, since dog urine had a lower protein concentration, and larger volumes were available, protein was first concentrated by chloroform and methanol precipitation, before resuspension in 0.5 M TRIS, 0.5% sodium dodecyl sulfate (SDS) buffer, pH 7. The protein concentration of this resuspension was calculated using the DC protein assay (Bio-Rad), as was pure mouse urine using the Micro Bradford assay (Bio-rad). After normalisation to protein content, samples were either diluted 1:1 in 12% SDS for carbonyl assay or in 2:1 in sample buffer containing 0.4 M TRIS pH 7, 6% SDS, 60% glycerol, 0.06% bromophenol blue and 10% dithiothreitol (DTT). Samples were then incubated at 100°C for 5 min for DiBrY and albumin labelling.

Frozen muscles (vastus lateralis for dogs and quadriceps for mice) were crushed using a mortar and pestle under liquid nitrogen. Muscle tissue was then homogenized in 1 ml ice-cold 1% NP40, 1 mM EDTA in phosphate buffered saline (PBS), supplemented with complete EDTA free protease inhibitor tablets (Roche), and centrifuged at 10000 *g* for 10 min. The protein concentrations of supernatants were quantified using the DC protein assay (Bio-Rad). After normalisation of protein content to 1 mg/ml, samples were either diluted 1:1 in 12% SDS for carbonyl assay or diluted 1:1 in sample buffer containing 0.3 M TRIS, 3% SDS, 30% glycerol, 0.03% bromophenol blue and 5% DTT before incubation at 100°C for 5 min for DiBrY labelling.

Plasma samples were diluted in 50x 1% NP40, 1 mM EDTA in PBS. Samples were then either further diluted 1:1 in 12% SDS for DNP labelling or in 1:1 in sample buffer containing 0.3 M TRIS pH 7, 3% SDS, 30% glycerol, 0.03% Bromophenol Blue and 5% DTT before incubation at 100°C for 5 minutes for DiBrY labelling.

Before resolution, samples for DNP labelling were mixed 1:1 with 10 mM 2,4-dinitrophenylhydrazine (DNPH) diluted in 10% trifluoroacetic acid. After incubation for 15 minutes, samples were mixed with 2 M TRIS, 30% glycerol, 0.05% bromophenol blue and 7.5% DTT before incubation at 100°C for 5 min. Plasma samples were further diluted four more times in the above samples buffer (without DTT) prior to loading.

Samples were resolved on 4–15% SDS-PAGE TGX Stain-Free gels (Bio-Rad) and total protein quantified using the Stain-Free imaging program on the ChemiDoc MP Imaging System (Bio-Rad). Gels were transferred onto nitrocellulose membrane using a Trans Turbo Blot system (Bio-Rad). Immunoblotting was performed with rabbit whole antiserum antibodies to DNP (D9656, Sigma) diluted 1:25000 in 5% skim milk in TRIS buffered saline with Tween (TBST), mouse monoclonal antibodies to di-halogenated tyrosine (DiBrY, NNS-MBY-020P, Cosmo Bio Co) diluted 1:1000 in 5% bovine serum albumin in TBST and anti-rabbit albumin (49296, Cell Signal) diluted 1:1000 in TBST. HRP-conjugated goat anti-rabbit secondary antibodies were from Thermo Fisher Scientific diluted 1:25000 in 5% skim milk in TBST and HRP-conjugated sheep anti-mouse secondary antibodies were from GE Healthcare, diluted 1:10000 in 5% skim milk in TBST. Chemiluminescence signal was captured using the ChemiDoc MP Imaging System (Bio-Rad). Resultant images were quantified using ImageJ software [40]. Stain-Free images were used as loading controls where applicable.

### Identification of DiBrY labelled protein in GRMD urine via mass spectrometry

The strongest DiBrY labelling was observed on a non-abundant protein in dog urine. In order to identify this protein, urine samples were run on two-dimensional SDS-PAGE followed by immunoblotting and mass spectrometry. Urine was first concentrated by chloroform and methanol precipitation, before resuspension in 7 M urea, 2 M thiourea, 4% CHAPS and 40 mM TRIS. Ampholyte (1%) and DTT were added before focusing on 7 cm ReadyStrip IPG Strips, pH 3–10 (Bio-Rad) using the IPGphor Isoelectric Focusing System (Amersham Pharmacia Biotech). After focusing, gel strips were run onto 10% acrylamide gels. One gel was immunoblotted with DiBrY, as per above, and one stained with Coomassie brilliant blue. Membrane images were overlaid on gel images, and the protein that was labelled by DiBrY was excised from the gel for mass spectrometry.

The gel spot was cut into 1 mm cubes and de-stained 3 times with 100 μl of 25 mM ammonium bicarbonate in 50% acetonitrile (ACN) at 37°C for 30 min followed by vacuum centrifugation. Protein was digested with 150 ng trypsin in 25 mM ammonium bicarbonate. The digestion reaction proceeded at 37°C for 16 hours. Digested protein was extracted by three additions of 1% triflouroacetic acid in can, incubated at room temperature for 20 min, and then desiccated by vacuum centrifugation [13].

The extract was reconstituted in 2% ACN, 0.1% formic acid (FA) solution for loading into a Prominence (Shimadzu) HPLC for chromatographic separation, which was directly sprayed into a 5600 TripleTOF^TM^ (Sciex) mass spectrometer. The HPLC mobile phase consisted of 0.1% FA and 2% ACN in water (A) and 0.1% FA and 2% water in ACN (B). Gradient elution was performed with 2% B for 3 min, increased to 40% B at 15 min, then ramped to 98% B by 16 min and held for 2 min before reduced back to 2% B within 1 min. Column temperature was 40°C [13].

Positive electrospray ionization mode was operated to acquire MS data by information-dependent acquisition (IDA), where only the top 20 MS peaks between 400 and 1250 m/z were selected for further MS/MS scan. Mass tolerance was 50 mDa. BSA calibration was conducted before a batch of samples was run. MS/MS data was imported into the database search engine Mascot (www.matrixscience.com) for identification of peptides and proteins, using the following search conditions: Swissprot database, all mammalian species, trypsin digest with allowance for up to one missed cleavage per peptide, no fixed modifications, variable modification of oxidation on methionine residues, MS tolerance of 0.2 Da, MS/MS tolerance of 0.2 Da. Protein identification was determined on the basis of 2 or more identified peptides with ion scores exceeding the significance threshold. The protein spot was identified as being serum albumin (canine), accession number P49822, with 14 significant peptides.

### Normalisation

While analytes in urine are generally normalized to creatinine content, since the methods measure modification to specific proteins, rather than content of specific proteins, it was deemed more suitable to standardise signal from the DNP and DiBrY antibodies to signal of that specific protein where possible, and to total protein when multiple proteins were labelled. Specifically, since labelling of DNP and DiBrY occurred on highly abundant albumin in plasma and major urinary proteins (MUP) in mouse urine, the signal was standardised to signal of that protein from the stain free images. Because labelling of DNP and DiBrY occurred on albumin in urine, the signal was standardised to albumin content, using an antibody for albumin. In muscle, many proteins were labelled and, thus, signal from whole lanes were analysed, and standardised to total protein for that lane using the stain free images. Since DiBrY labelling in dog urine was identified on a fragment of albumin (discussed below), signal was standardised to total albumin, as the fragment was not detectable by immunoblotting.

### Plasma creatine kinase (CK) and muscle myeloperoxidase (MPO) levels

Plasma CK activity was determined using the CK-NAC kit (Ran-dox Laboratories) and analyses kinetically using a BioTekPowerwave XS Spectrophotometer using the KC4 (v 3.4) program.

MPO catalyses the production of hypochlorous acid from hydrogen peroxide and chloride and hypochlorus acid reacts with 2-[6-(4-aminophenoxy)-3-oxo-3H-xanthen-9-yl]benzoic acid (APF) to form the highly fluorescent compound fluorescein, that is measured in this method, as per [14]. Briefly, muscle was ground using a mortar and pestle under liquid nitrogen and homogenised in 0.5% hexadecyltrimethylammonium bromide in PBS. Samples were centrifuged and supernatants diluted in PBS. Human MPO was used as the standard for the assay (Cayman Chemical). Aliquots of each experimental sample or MPO standard was pipetted into a 384 well plate, before the addition of APF working solution (20 μM APF and 20 μM hydrogen peroxide in PBS) was added. The plate was incubated at room temperature (protected from light) for 30 minutes, with the fluorescence being measured every minute using excitation at 485 nm and emission at 515–530 nm. The rate of change of fluorescence for each sample was compared to that of the standards and results were expressed per mg of protein, quantified using the DC protein assay (Bio-Rad).

### Statistics

Data were analysed using GraphPad Prism software. Unpaired parametric T-tests were used to identify significant differences between GRMD and healthy dog tissues, and mdx and C57 tissues. Differences between strains were not analysed due to differences in immunoreactivity. Statistical significance was accepted at p<0.05. All data points are presented ± SEM and n = 5–7.

## Results

### Protein identifications of urinary proteins undergoing carbonylation and tyrosine modifications

In GRMD urine, DNP labelling occurred on multiple proteins, with a strong signal from the highly abundant albumin (whole lane shown in S2a). Therefore, analyses were performed on signal from both the whole lane and albumin. In GRMD urine, DiBrY labelling occurred on a protein of approximately 50 kDa (whole lane shown in S2b). The protein was identified as a fragment of albumin by mass spectrometry. In mdx urine, DNP labelling was observed on the highly abundant albumin, and MUP (whole lane shown in S2c,). In mdx urine, DiBrY labelling was observed on the highly abundant MUP (whole lane shown in S2d). In both GRMD and mdx muscle, DNP and DiBrY labelling was observed on numerous proteins (an example whole lane shown in S2e, DNP labelling of GRMD muscle). In both GRMD and mdx plasma, DNP and DiBrY labelling was observed mainly on albumin (an example whole lane shown in S2f, DNP labelling of mdx plasma).

### GRMD urine: Carbonylated proteins and protein tyrosine modifications

**Fig 1:**
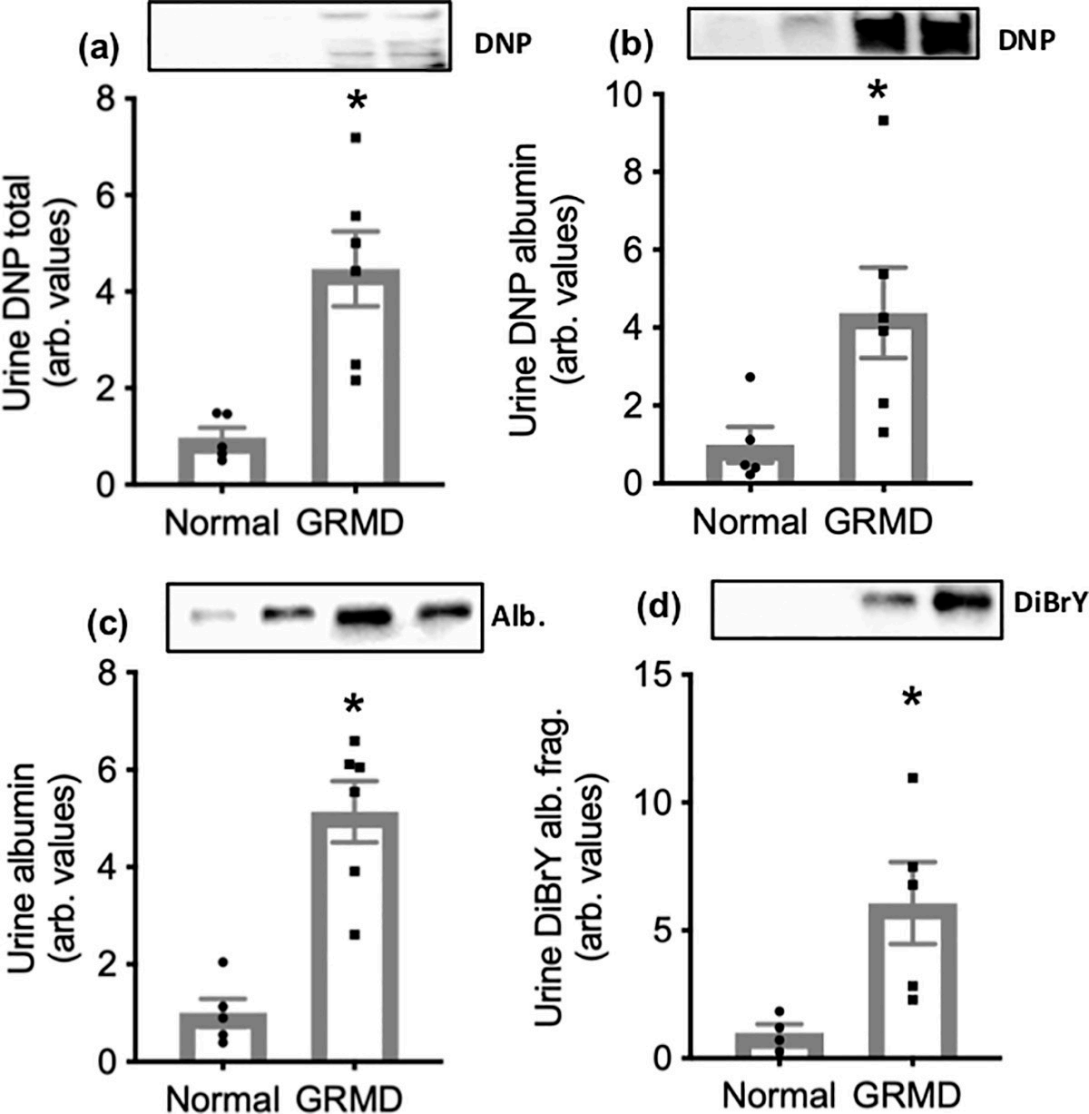
Total protein carbonylation (a), albumin carbonylation (b), albumin content (c) and protein tyrosine modifications (d) in urine from normal and GRMD dogs. Asterisks represent significant differences of p<0.05. Data are presented as mean ± SEM and n = 5 and 6 respectively. (a), (c) and (d) were standardised to total protein and (b) was standardised to albumin content. Representative blot images are shown of DNP labelling of several proteins bands (a) and of albumin (b), albumin protein content (c) and DiBrY labelling of several proteins bands (d).

Immunoblotting using DNP and DiBrY antibodies to determine protein oxidative damage was performed on urine from normal control and GRMD dogs. Analyses were performed on signal from both the whole lane and albumin as the predominantly oxidised protein. DNP labelling of total protein was nearly 3-fold higher in GRMD urine compared with normal dog urine (Fig 1A). DNP labelling of albumin (standardised to urine albumin content) was 3-fold higher in GRMD urine, compared with normal dog urine (Fig 1B). The content of albumin in urine was 1.7-fold higher in GRMD urine compared with healthy dog urine (Fig 1C). As discussed above, DiBrY labelling occurred on a fragment of albumin of approximately 50 kDa. However, this fragment was not immunoreactive to an albumin antibody, so we were unable to normalise to its content. Instead, DiBrY labelling in urine was normalised to total albumin content, and was 6-fold higher in GRMD urine compared with healthy dog urine (Fig 1D).

### Mdx urine: Carbonylated proteins and protein tyrosine modifications

**Fig 2:**
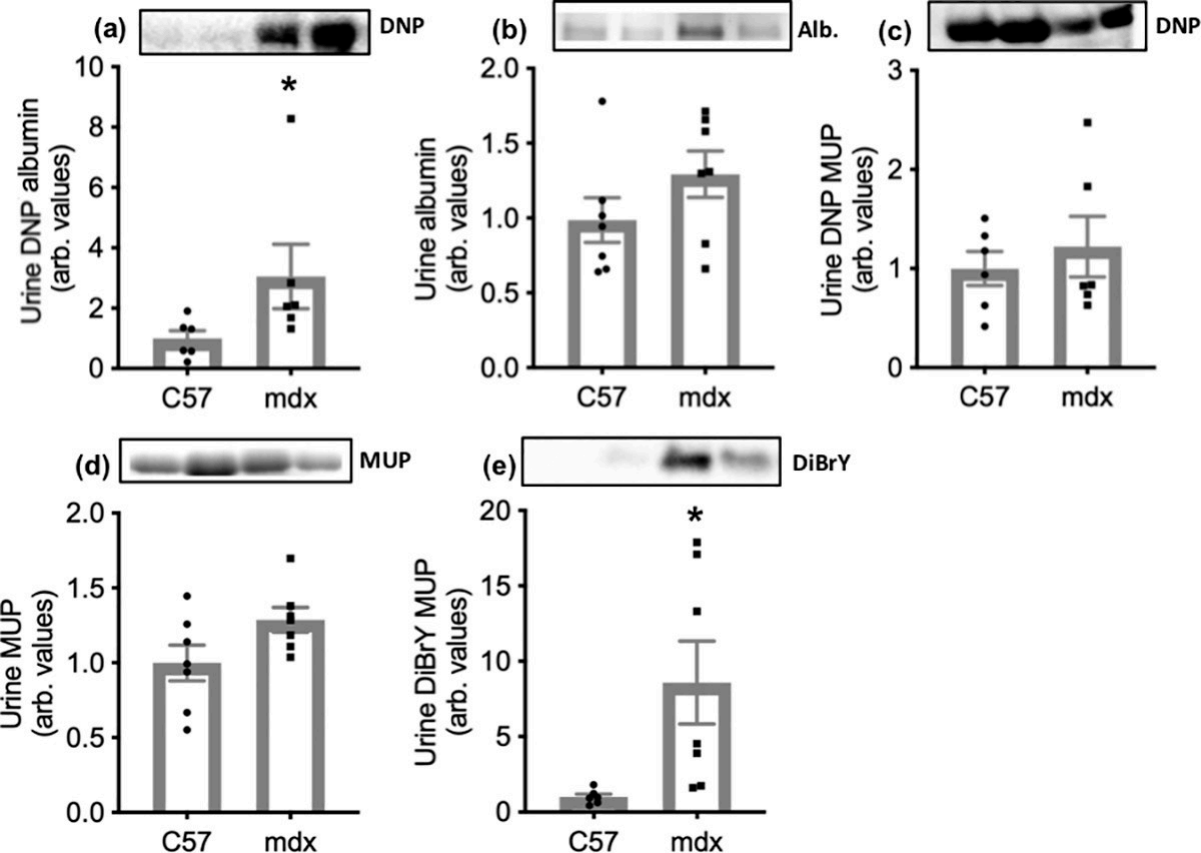
Albumin carbonylation (a), albumin content (b), MUP carbonylation (c), MUP content (d) and MUP tyrosine modifications (e) in urine from C57 and mdx mice. Asterisks represent significant differences of p<0.05. Data are presented as mean ± SEM and n = 6 and 7 respectively. (a) was standardised to albumin content, (b) and (f) were standardised to total protein and (c) and (e) were standardised to MUP content. Representative blot images are shown of DNP labelling of albumin (a), albumin protein content (b), DNP labelling of MUP (c), MUP protein content (d), and DiBrY labelling of MUP (e).

For urine from C57 control and mdx mice, DNP labelling was predominantly observed on albumin and MUP in mdx urine. DNP labelling of albumin (standardised to albumin content) was 3-fold higher in mdx urine compared with C57 (Fig 2A). There was no difference in excretion of albumin (Fig 2B), DNP labelling, or excretion of MUP between mdx and C57 urine samples (Fig 2C and 2D). DiBrY labelling was only observed on the MUP in mdx urine. The DiBrY labelling of MUP (standardised to MUP content) was nearly 9-fold higher in mdx compared with C57 urine (Fig 2E).

### GRMD and mdx muscle: Carbonylated proteins and protein tyrosine modifications

**Fig 3:**
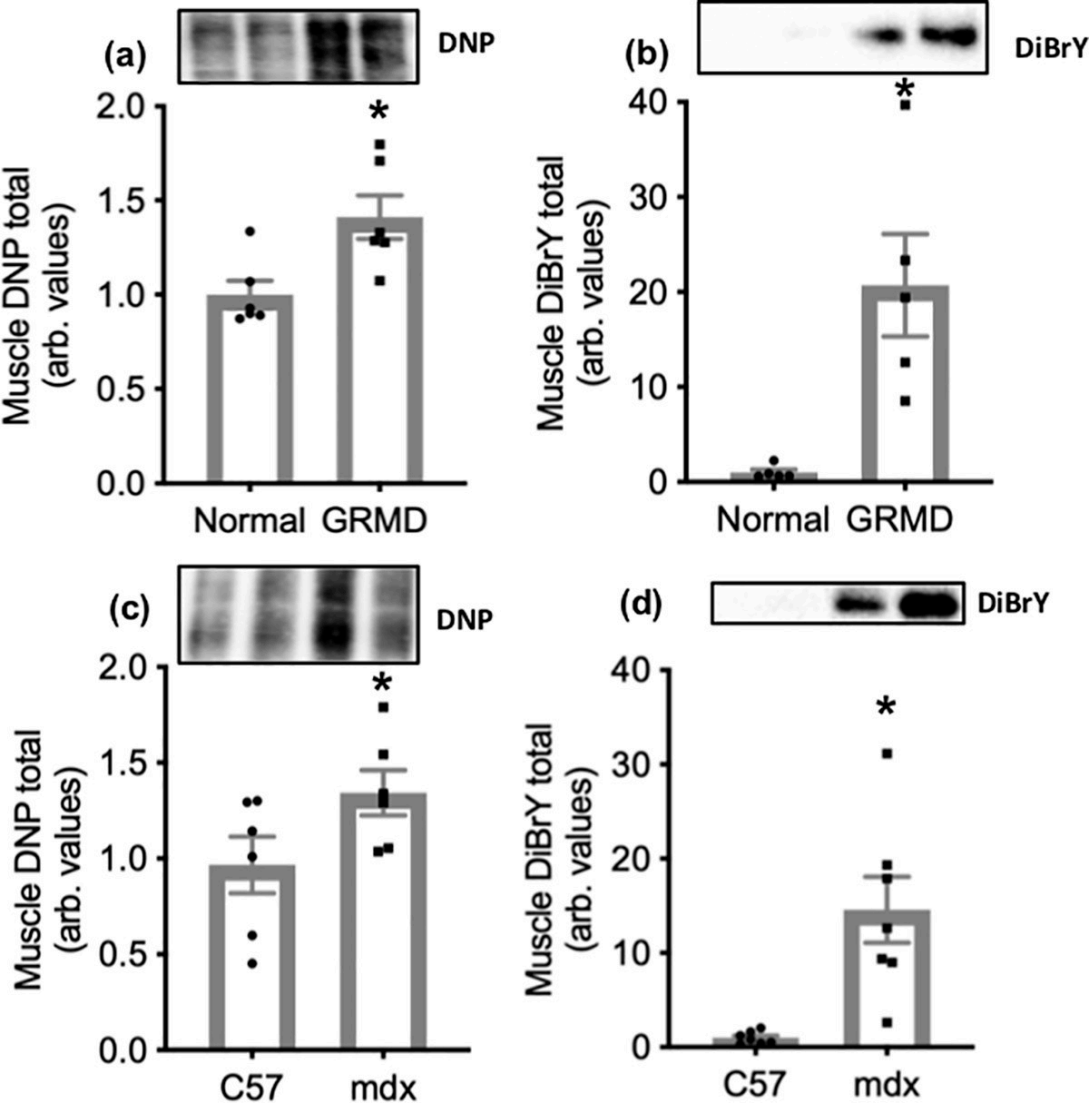
Total protein carbonylation and protein tyrosine modifications (B) in muscle from normal and GRMD dogs (a and b respectively) and C57 and mdx mice (c and d respectively). Asterisks represent significant differences of p<0.05. Data are presented as mean ± SEM and n = 6. All were standardised to total protein. Representative blot images are shown of DNP labelling of several proteins’ bands (a and c) and DiBrY labelling of predominately labelled proteins (b, approximately 50 kDa and d, approximately 25 kDa).

DNP and DiBrY labelling were 1.4-fold and 20-fold higher in GRMD muscle compared with normal dogs, respectively (Fig 3A and 3B). DNP and DiBrY labelling were respectively 1.3-fold and 15-fold higher in mdx muscle compared with C57 (Fig 3C and 3D).

### GRMD and mdx plasma: Carbonylated proteins and protein tyrosine modifications

**Fig 4:**
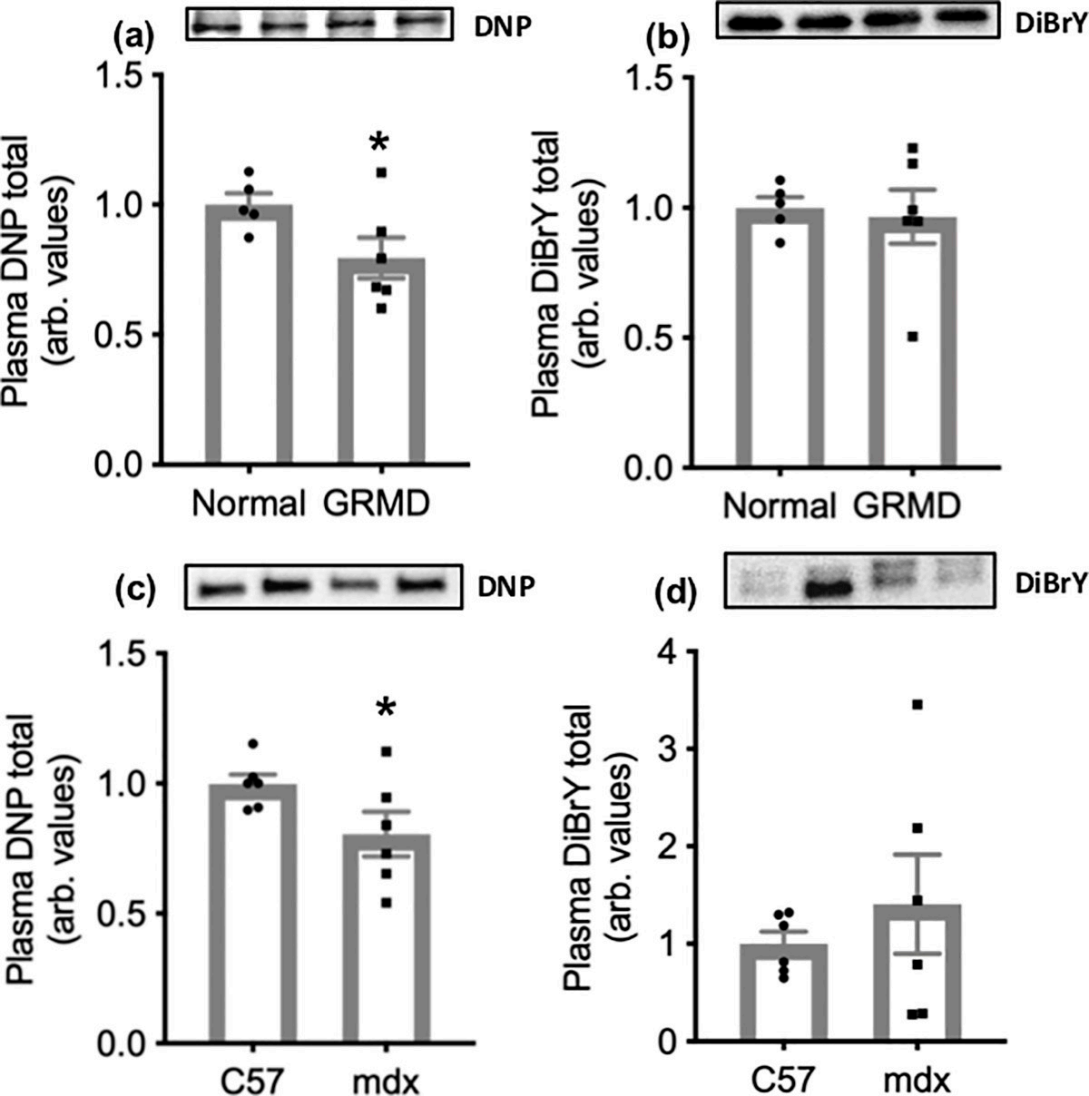
Albumin carbonylation and protein tyrosine modifications in plasma from normal and GRMD dogs (a and b respectively) and C57 and mdx mice (c and d respectively). Asterisks represent significant differences of p<0.05. Data are presented as mean ± SEM and n = 6. (a) is standardised to albumin content and (b) was standardised to total protein. Representative blot images are shown of DNP labelling of albumin (a and c) and DiBrY labelling of predominately labelled proteins (b and d, approximately 50 kDa).

DNP labelling was observed only on albumin in GRMD plasma; therefore, only data for carbonylation of albumin are presented. DNP labelling of albumin was 30% lower in GRMD compared with normal plasma (Fig 4A). There was no difference in DiBrY labelling between GRMD and normal dog plasma (Fig 4B). DNP labelling was observed only on albumin in mdx plasma; therefore, only data for carbonylation of albumin are presented. DNP labelling was 20% lower in mdx compared with C57 plasma (Fig 4C). There was no difference in DiBrY labelling between mdx and C57 plasma (Fig 4D).

### Correlation between muscle and urine protein oxidation makers, and measures of dystropathology

To explore the relationship between muscle and blood measurements of protein oxidation, as well as the relationship between these measures and an established biomarker of dystropathology, plasma CK, and neutrophil presence in muscle (MPO), correlation analysis was performed in dogs (Table 1). CK was 200-fold higher in GRMD plasma compared with normal dogs (3571±177 and 16±4.5 units respectively). MPO was 9-fold higher in GRMD muscle compared with normal dogs (0.28±0.06 and 0.04±0.005 nmole^-1^ per mg protein^-1^ respectively). A significant positive relationship was observed between urine and muscle DiBrY, urine DiBrY and plasma CK, urine DiBrY and muscle MPO, muscle DiBrY and plasma CK and muscle DiBrY and muscle MPO (Table 1). No relationship was observed between urine DNP and muscle DNP (Table 1), however a significant positive relationship was observed between urine DNP and plasma CK, muscle DNP and plasma CK and muscle DNP and muscle MPO.

**Table 1:** Correlation between muscle and urine protein oxidation makers, and measures of dystropathology (plasma CK) and muscle neutrophil presence (MPO) in dogs.

Indices 1	Indices 1	*r*	*p*
Urine DiBrY	Muscle DiBrY	0.9594	<0.0001*
Urine DiBrY	Plasma CK	0.8585	0.0030*
Urine DiBrY	Muscle MPO	0.8990	0.0010*
Muscle DiBrY	Plasma CK	0.8432	0.0022*
Muscle DiBrY	Muscle MPO	0.9440	<0.0001*
Urine DNP	Muscle DNP	0.5557	0.0759
Urine DNP	Plasma CK	0.7959	0.0059*
Urine DNP	Muscle MPO	0.6156	0.0581
Muscle DNP	Plasma CK	0.6886	0.0277*
Muscle DNP	Muscle MPO	0.8036	0.0051*

N = 12 and asterisks represent significant correlation of p<0.05.

## Discussion

Our key observation is that, compared with healthy animals, increased levels of urinary proteins were detected with antibodies to both DNP and DiBrY in dystrophic GRMD dogs and mdx mice; a consequence of oxidative damage in muscle and modifications by the highly reactive oxidants. DiBrY labelling (specific for di-halogenated tyrosine formed directly by myeloperoxidase generated oxidants) of urine and muscle proteins showed a striking difference between dystrophic strains and controls. A strong positive correlation between oxidation to proteins in urine and muscle (DiBrY) was observed in dogs. A strong positive correlation was also observed when comparing DNP and DiBrY labelling (in muscle and urine) to markers of dystropathology (plasma CK) and neutrophil presence (muscle MPO). These data support the potential use of urine DiBrY as a biomarker for tracking neutrophil mediated oxidative stress and dystropathology in DMD. Of note, the proteins predominantly labelled by DiBrY differed between urine in GRMD dogs and mdx mice which will need to be considered for any comparisons of neutrophil activity across species.

We and others have previously demonstrated that neutrophil elastase and protein carbonylation are elevated in mdx, GRMD and DMD muscle [13, 21–24]. In this study, we further expanded upon our previous data and showed increased carbonylation of urinary proteins in GRMD dogs and mdx mice, reflecting an increase in muscle protein carbonylation in both species. Many different proteins exhibit carbonylation in GRMD and mdx muscle and urine, with albumin exhibiting the strongest signal. While carbonylation of urinary proteins has not been previously shown in DMD or its animal models, a similar increase in urine proteins has been seen in humans after competing in super-marathons or undertaking an over-training resistance protocol and in patients with chronic renal failure [29–31]. Interestingly, in patients with chronic renal failure, carbonylation of urinary proteins and specifically albumin was 99% and 71% higher, respectively, compared with plasma [31]. Likewise, in the current study, both species of dystrophic animals showed increased carbonylation of urinary proteins, but a decrease in carbonylation of plasma albumin. It is not clear why in GRMD dogs and mdx mice there is a difference in carbonylation of plasma and urinary proteins. Although it is possible that there is increased glomerular filtration of oxidised proteins, effectively removing them from circulation, there is limited experimental research that supports this concept. However, in humans with kidney disease, acute oxidative stress leads to a transient increase in protein excretion, including increased albumin excretion [41]. Likewise, in the current study, GRMD urine showed increased excretion of total albumin, supporting the concept that oxidised proteins are more readily excreted and potentially also suggesting some kidney dysfunction in dystrophic dogs. While kidney dysfunction is not widely reported in DMD patients and animal models, myoglobinuera has been shown, which could lead to acute tubular necrosis [42]. Further experimental work is required to understand the excretion of oxidatively damaged proteins in GRMD dogs and mdx mice, and albuminuria in GRMD dogs. Regardless, the carbonylation of proteins in the urine of these dystrophic animals has the potential to be useful as biomarkers of oxidative stress.

We have previously shown increased neutrophil content, HOCl generation and associated oxidative stress in muscles from GRMD dogs and mdx mice [13, 14], leading us to hypothesise that oxidants in dystrophic DMD muscle are generated in large part by highly reactive oxidant hypochlorous acid (HOCl) in neutrophils. In this study, consistent with our hypothesis, we show that proteins in GRMD and mdx urine undergo protein tyrosine modifications (the formation of di-halogenated tyrosine, as shown by an increase in DiBrY labelling), indicating an increase in exposure to highly reactive oxidants, such as HOCl, generated by myeloperoxidase [11]. The dystrophic muscle from both models also showed a dramatic increase in DiBrY labelling, which had a strong correlation to urine levels. Increased DiBrY labelling of both urine and muscle proteins also correlated strongly with muscle MPO, supporting the use of the measure as a biomarker of neutrophil mediated oxidative stress. Interestingly, increased protein tyrosine modifications were not detected in either GRMD nor mdx plasma. While the modification of urinary protein tyrosines has not been previously reported in DMD nor animal models for DMD, they have been shown in urine of aging rats, correlating with tyrosine residue modification in skeletal muscle [43], and in urine from diabetic patients [44]. In this study, multiple proteins underwent tyrosine halogenation in GRMD and mdx muscle, but tyrosine halogenation in urine appeared to occur primarily only on fragmented albumin in GRMD and MUP (major urinary proteins) in mdx. Using mass spectrometry, we identified that the protein undergoing halogenation in GRMD urine was albumin, specifically a fragment of approximately 50 kDa (since the molecular mass of the protein was smaller than intact albumin at 68 kDa). Albumin is rich in tyrosines, which are extremely reactive with HOCl [45]. In urine, a significant amount of albumin exists in fragments, representing 20–30% of the urinary protein pool in normal conditions [45]. Fragments are formed by proteolytic processes during circulation and in the urinary tract [46] and likely represent breakdown products of albumin. Very little research exists on the oxidation of specific fragments of albumin; however, a highly reactive tyrosine residue has been reported in a fragment of human albumin [47]. Likewise, the fragment of albumin found in our study is highly susceptible to tyrosine halogenation. It is also not known if halogenation of tyrosine in fragmented albumin occurs prior to or after fragmentation, or whereabouts it occurs. For example, albumin or the fragment could be exposed to HOCl during passage through the muscle, or it could be a consequence of systemic inflammation.

Interestingly, mdx urine exhibited tyrosine halogenation on MUP, which are one of the most abundant proteins in mouse urine. MUP bind and release small volatile pheromones as scent marks, signalling the identity of the mouse [48]. A literature search did not identify any previous research into the modifications of MUP by halogenation of tyrosine residues. Certain MUPs have been shown to be rich in tyrosines, likely making them sensitive to halogenation [49]. On the other hand, MUP in dog urine did not exhibit halogenation, suggesting perhaps the proteins do not have equal sensitivity to reactive oxidants generated by myeloperoxidase. Like halogenation of albumin or its fragment, it is not known where mdx MUP are exposed to reactive oxidants. One possibility is that during blood flow through the inflamed dystrophic muscle, circulating MUP are exposed to reactive oxidants.

We found increased oxidised proteins using two methods (DNP and DiBrY immunolabelling) in GRMD and mdx urine, reflecting the oxidative state of muscle from both dystrophic species. This was also supported by the strong positive correlation between muscle and urine DiBrY labelling in GRMD dogs. Given the relative ease of urine collection, measuring the extent to which proteins are oxidised in urine have the potential to be useful biomarkers in monitoring disease progression, or the response to a putative treatment in preclinical drug trials. DiBrY labelling of urine proteins showed a striking difference between dystrophic animals and controls, more so than the DNP labelling (which is a general measure of oxidative damage). The presence of halogenated proteins enables tracking of neutrophil mediated oxidative stress, and in this context, immunoassays using DiBrY labelling targeting specific proteins have the potential to be very useful, as the techniques are widely accessible and can be modified into high throughput ELISAs.

## Supporting information

S1 Fig Immunoreactivity of plasma treated with HOCl, hydrogen peroxide (H202) and hydroxl radical (•OH) using DNP and DiBrY antibodies. (TIF)

S2 Fig DNP and DiBrY blots, showing urinary proteins undergoing carbonylation and tyrosine modification. Images show two representative images of protein content and equivalent immunoassay blot in urine; also included are examples of protein content and immunoblotting of muscle and plasma. (a) GRMD urine, with DNP labelling on multiple proteins, with the predominant signal from albumin (68 kDa). (b) GRMD urine, with DiBrY labelling on a fragment of albumin of approximately 50 kDa. (c) mdx urine, with DNP labelling on albumin (68 kDa), and MUP (19 kDa). Imaging of each protein was performed at different intensities, so the image is a composite. (d) mdx urine, with DiBrY labelling on MUP (19 kDa). (e) GRMD muscle, with DNP labelling on numerous proteins (arrows). (f) mdx plasma, showing DNP labelling on albumin.
(TIF)

S1 Raw images Original blot contained in figures. Dystrophic animals (GRMD and mdx) are annotated with *, others are healthy/wildtype controls.
(PDF)
