# Supplementary figures and images for: Oxidative damage to urinary proteins from the GRMD dog and mdx mouse as biomarkers of dystropathology in Duchenne muscular dystrophy
Source: PLoS One. 2020 Oct 8;15(10):e0240317. doi: 10.1371/journal.pone.0240317 (PMC7544076; doi:10.1371/journal.pone.0240317)

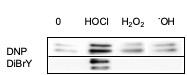

Supplement: S1 Fig — (TIF) [file pone.0240317.s001.tif]

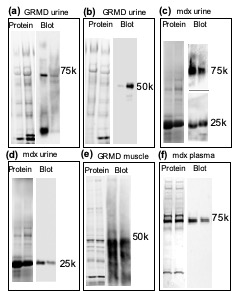

Supplement: S2 Fig — Images show two representative images of protein content and equivalent immunoassay blot in urine; also included are examples of protein content and immunoblotting of muscle and plasma. (a) GRMD urine, with DNP labelling on multiple proteins, with the predominant signal from albumin (68 kDa). (b) GRMD urine, with DiBrY labelling on a fragment of albumin of approximately 50 kDa. (c) mdx urine, with DNP labelling on albumin (68 kDa), and MUP (19 kDa). Imaging of each protein was performed at different intensities, so the image is a composite. (d) mdx urine, with DiBrY labelling on MUP (19 kDa). (e) GRMD muscle, with DNP labelling on numerous proteins (arrows). (f) mdx plasma, showing DNP labelling on albumin. (TIF) [file pone.0240317.s002.tif]
