# Supplementary material for: Oxidative damage to urinary proteins from the GRMD dog and mdx mouse as biomarkers of dystropathology in Duchenne muscular dystrophy
Source: PLoS One. 2020 Oct 8;15(10):e0240317. doi: 10.1371/journal.pone.0240317 (PMC7544076; doi:10.1371/journal.pone.0240317)

Dystrophic animals (GRMD and mdx) are annotated with *, others are healthy/wildtype controls
All images are captured with ECL using Bio-rad Chemidoc technology

Urine membranes

Dog albumin (FIG 1C)

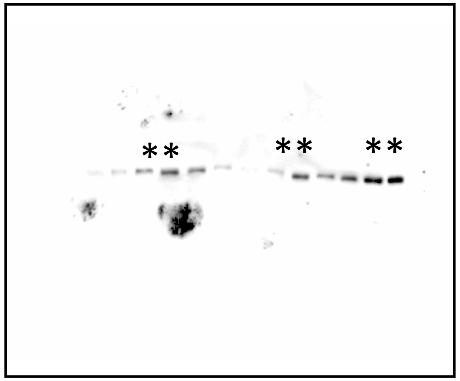

Dog DNP (FIG 1A)

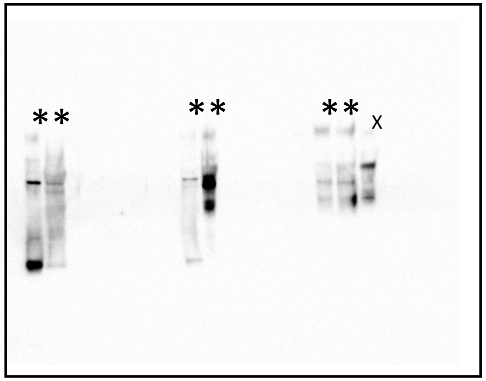

Dog DiBrY (FIG 1D)

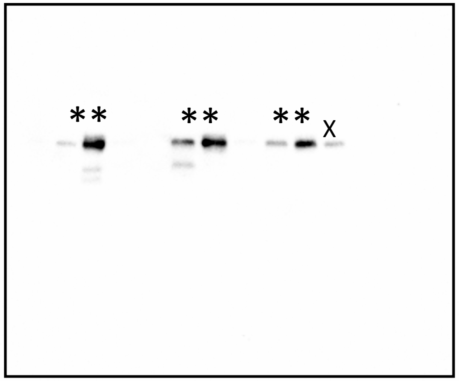

Mouse DNP alb (FIG 2A)

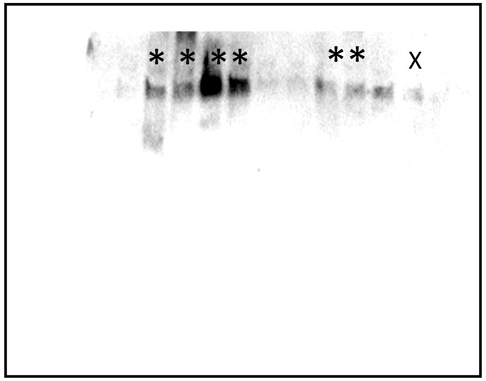

Mouse DNP MUP (FIG 2C)

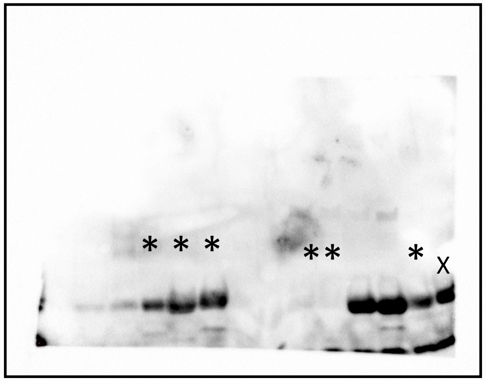

Mouse DiBrY MUP (FIG 2E)

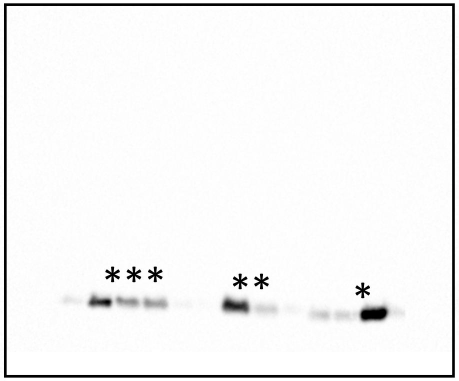

Muscle membranes

Dog DNP (FIG 3A)

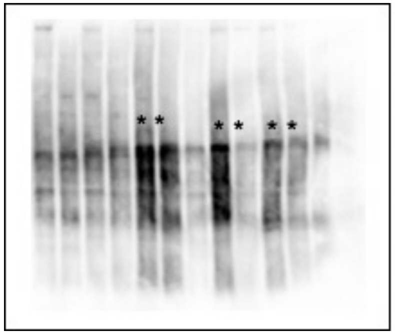

Dog DiBrY (FIG 3B)

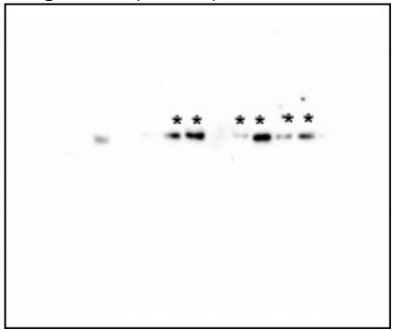

Mouse DNP (FIG 3C)

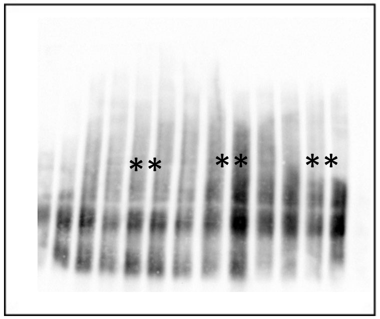

Mouse DiBrY (FIG 3D)

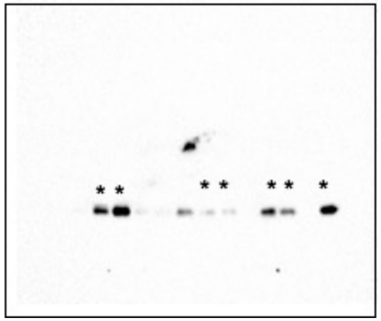

Plasma membranes

Dog DNP (FIG 4A)

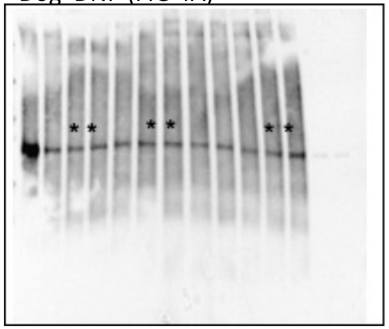

Dog DiBrY (FIG 4B)

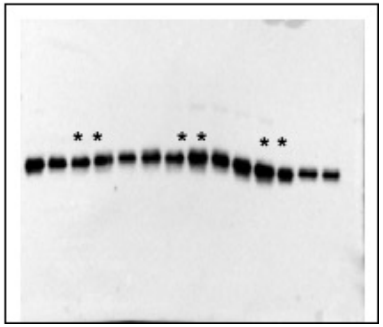

Mouse DNP (FIG 4C)

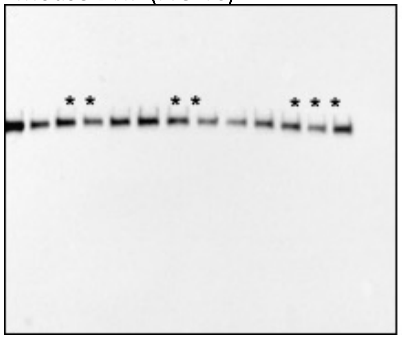

Mouse DiBrY (FIG 4D)

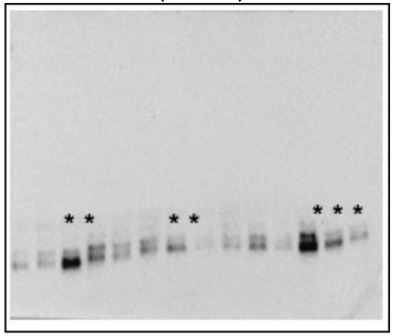

Supplement: S1 Raw images — Dystrophic animals (GRMD and mdx) are annotated with *, others are healthy/wildtype controls. (PDF) [file pone.0240317.s003.pdf]
